# The influence of measurement error on calibration, discrimination, and overall estimation of a risk prediction model

**DOI:** 10.1186/1478-7954-10-20

**Published:** 2012-11-01

**Authors:** Laura C Rosella, Paul Corey, Therese A Stukel, Cam Mustard, Jan Hux, Doug G Manuel

**Affiliations:** 1 Public Health Ontario, Toronto, Ontario, Canada; 2 Dalla Lana School of Public Health, University of Toronto, Toronto, Ontario, Canada; 3 Institute for Clinical Evaluative Sciences, Toronto, Ontario, Canada; 4 Department of Health Policy, Management, and Evaluation, University of Toronto, Toronto, Ontario, Canada; 5 Institute of Work and Health, Toronto, Ontario, Canada; 6 Ottawa Hospital Research Institute, Ottawa, Ontario, Canada; 7 Statistics Canada, Ottawa, Ontario, Canada

## Abstract

**Background:**

Self-reported height and weight are commonly collected at the population level; however, they can be subject to measurement error. The impact of this error on predicted risk, discrimination, and calibration of a model that uses body mass index (BMI) to predict risk of diabetes incidence is not known. The objective of this study is to use simulation to quantify and describe the effect of random and systematic error in self-reported height and weight on the performance of a model for predicting diabetes.

**Methods:**

Two general categories of error were examined: random (nondirectional) error and systematic (directional) error on an algorithm relating BMI in kg/m^2^ to probability of developing diabetes. The cohort used to develop the risk algorithm was derived from 23,403 Ontario residents that responded to the 1996/1997 National Population Health Survey linked to a population-based diabetes registry. The data and algorithm were then simulated to allow for estimation of the impact of these errors on predicted risk using the Hosmer-Lemeshow goodness-of-fit χ^2^ and C-statistic. Simulations were done 500 times with sample sizes of 9,177 for males and 10,618 for females.

**Results:**

Simulation data successfully reproduced discrimination and calibration generated from population data. Increasing levels of random error in height and weight reduced the calibration and discrimination of the model. Random error biased the predicted risk upwards whereas systematic error biased predicted risk in the direction of the bias and reduced calibration; however, it did not affect discrimination.

**Conclusion:**

This study demonstrates that random and systematic errors in self-reported health data have the potential to influence the performance of risk algorithms. Further research that quantifies the amount and direction of error can improve model performance by allowing for adjustments in exposure measurements.

## Introduction

In medicine, prediction tools are used to calculate the probability of developing a disease or state in a given time period. Within the clinical setting, predictive algorithms, such as the Framingham Heart Score 31 [1] are used to calculate the probability that a patient will develop coronary heart disease – have contributed important advances in individual patient treatment and disease prevention [2]. Similarly, applying predictive risk tools to populations can provide insight into the influence of risk factors, the future burden of disease in an entire region or nation, and the value of interventions at the population level. Risk prediction is a key aspect of clinical work and has recently been applied to population health through the Diabetes Population Risk Tool (DPoRT) [3]. The prediction of disease risk using risk algorithms is based on a set of baseline variables that may contain measurement error that could affect the prediction, discrimination, and accuracy of the tool.

Increasingly, prediction tools have incorporated self-reported patient information to facilitate their use [3-5]. These self-reported responses can contain random error due to imperfect recall or misunderstanding of the question. They can also result in systematic error or bias (over- or underreporting), as a result of psychosocial factors such as social desirability. The influence of error contained in self-reported risk factor data on disease prediction has not been systemically studied. In particular, evidence on the influence that measurement error has on predictive accuracy is lacking. By understanding the consequence of measurement error on risk algorithms, efforts could be made to correct for these errors and thus improve the accuracy and validity of risk algorithms. Furthermore, developers of risk tools can use this information to better weigh the pros and cons of using different types of data (i.e., self-reported or measured).

Measurement error has mainly been examined with respect to its effect on risk estimates, such as risk ratios or hazard ratios [6-8]. This research has led to improvements in the critical appraisal and interpretation of epidemiologic findings. While useful for understanding the effects of error on etiological estimates of disease, the findings from these studies do not directly apply to risk algorithms. The objective of this study is to use simulation to understand the effect of measurement error in self-reported risk factors on the performance of a simple risk algorithm to predict diabetes. This study will focus on the measurement of body mass index (BMI), which is defined as an individual’s body mass in kilograms (kg) divided by the square of the individual’s height in meters (m) (kg/m^2^). This measure is the focus because it has the greatest influence on diabetes risk [9-13].

## Methods

Two general categories of error were examined in this study: random (nondirectional) error and systematic (directional) error. Data were simulated to allow for estimation of the impact of hypothetical values of random and systematic error on predicted risk and two measures of predictive accuracy: calibration and discrimination. Calibration is achieved in a prediction model if it is able to predict future risk with accuracy such that the predicted probabilities closely agree with observed outcomes. A model that does not have good calibration will result in a significant over- or underestimation of risk. Calibration is not an issue if the purpose of the model is only to rank-order subjects [14]. In this study, calibration was measured using the Hosmer-Lemeshow (H-L) goodness-of-fit statistic (χ^2^_H-L_) where observed and expected values are compared across deciles of risk [15-17]. It is calculated by dividing the cohort into deciles of predicted risk and comparing observed versus predicted risk resulting in a modified version of H-L chi-square statistic (χ^2^_H-L_). Consistent with D’Agostino’s approach for evaluating observed and predicted values using risk algorithms, the value 20 (99^th^ percentile of a chi-square with 8 degrees of freedom) was used as a cutoff to mark sufficient calibration [18]. Discrimination is the ability to differentiate between those who are high risk and those who are low risk – or, in this case, those who will and will not develop diabetes given a fixed set of variables. The receiver operating characteristic (ROC) curve is the accepted way to measure discrimination. An ROC curve repeats all possible pairings of subjects in the sample who exhibit the outcome and do not exhibit the outcome and calculates the proportion of correct predictions, thereby resulting in an index of resolution. This area under the ROC curve is equal to the C-statistic where 1.0 implies perfect discrimination and 0.5 implies no discrimination [14,19,20]. A perfect prediction model would perfectly resolve the population into those who develop diabetes and those who do not. Accuracy is unaffected by discrimination, meaning a model can possess good discrimination yet poor calibration [21].

The simulation was initiated using parameters taken from the same population-level data used to develop DPoRT [3]. These data represent 23,403 Ontario residents that responded to the 1996/1997 National Population Health Survey (NPHS) conducted by Statistics Canada [22] and were linkable to health administrative databases in Ontario. In the NPHS, households were selected though stratified, multilevel cluster sampling of private residences using provinces and/or local planning regions as the primary sampling unit. The survey was conducted by telephone and all responses were self-reported (83% response rate). Persons under the age of 20 (n = 2, 407) and those who had self-reported diabetes were excluded (n = 894). Those who were pregnant at the time of the survey were also excluded (n = 241) due to the fact that baseline BMI could not be accurately ascertained, leaving a total of 9,177 males and 10,618 females. The diabetes status of all respondents in Ontario was established by linking persons to the Ontario Diabetes Database (ODD), which contains all physician-diagnosed diabetes patients in Ontario identified since 1991. The database was created using hospital discharge abstracts and physician service claims. The ODD has been validated against primary care health records and demonstrated excellent accuracy for determining incidence and prevalence of diabetes in Ontario (sensitivity of 86%, specificity of 97%) [23,24].

Height and weight were assumed to come from a normal distribution with mean and standard deviation equal to those from the derivation cohort shown in Table 1. Individual values were generated by multiplying the standard deviation to a random variable from the standard normal distribution. This value is then added or subtracted from the mean (depending on the random number generated) and replicated 9,177 times for males and 10,618 for females. The logistic model relating BMI and diabetes was estimated according to the following equation:

$$\begin{array}{ll} Logit(P_{i}) & =\beta_{0}+\beta_{1}BMI_{i}+\beta_{2}BMI_{i}^{2}\\ & =X_{i}\beta \end{array}$$

where *X*_*i*_*β={1,BMI*_*i*_*,BMI*_*i*_^*2*^*}* and *P*_*i*_ represents the probability of developing diabetes in 10 years:

$$P_{i}=P\left((,Y_{I}=|X_{i}\right)=\frac{\exp\left(X_{i}\beta \right)}{1+\exp\left(X_{i}\beta \right)}$$

**Table 1:** Starting values taken from 1996–1997 National Population Health Survey (NPHS) used in simulation

**Parameter**	**Males**	**Females**
**mean (standard deviation)**	**(N = 9,177)**	**(N = 10,618)**
Height (m)	1.768 (0.075)	1.627 (0.069)
Weight (kg)	81.624 (13.805)	64.761 (12.320)
BMI (kg/m^2^)	26.076 (3.995)	24.495 (4.586)
Correlation for height and weight (r_hw_)	r_hw_ = 0.475	r_hw_ = 0.311
10-year DM incidence	9.17%	7.35%

Using the generated values for the regression coefficients β_0_, β_1,_ and β_2,_ the probability of the person having diabetes was calculated for each individual. The coefficients in the algorithms remain constant for each calculation in order to replicate the current practice where the same risk equation is applied to all individuals.

We assumed that the observed variance of height and weight contain some level of error and therefore the observed variance can be separated into the true variance of the measurement (σ^2^_true_) in the population and the variance that can be attributed to measurement error (σ^2^_error_). Random measurement error was defined by the intraclass correlation coefficient (ICC) as an estimate of the fraction of the total measurement variance associated with the true variation among individuals [25,26]. Systematic error, which we refer to as bias in our study, is defined as the difference in observed height and weight from the true value (without measurement error). In our study, the bias was defined as an overestimation in height (0 to 3.0 cm) and an underestimation of weight (0 to −3.0 kg) varied in increments of 0.5 units. The magnitude of bias in height and weight were taken from a recent systematic review that summarized the empirical evidence regarding the concordance of objective and subjective measures of height and weight [27], consistent with those found in the Canadian population [28]. True BMI is defined as the height and weight when measurement error is equal to zero.

The simulation of each sample population was run 500 times. For each simulation *P*_*i*_ was calculated twice for each individual. The first calculation is done using the observed height and weight values and the second is related using the true BMI value (in the absence of the specified measurement error). H-L statistic and C-statistic were also calculated twice using both true BMI and observed BMI values to allow for comparison. All simulations were done using SAS statistical software (version 9.1, SAS Institute Inc., Cary, NC) and random numbers were generated using the RAN family of functions (RANUNI and RANNORM).

We had three a priori hypotheses prior to running the simulation. First, we hypothesized that random measurement error would affect both discrimination and calibration of a model due to the increase in observed variance in BMI and misclassification. Secondly, we hypothesized that systematic error would have minimal effects on discrimination (the ability to rank order subjects) but significant effects on calibration of a model. Thirdly, we hypothesized that random error would not affect the overall predicted risk value and that systematic error would influence the predicted risk in the direction of the systematic error.

## Results

To test the quality of the simulated data we estimated the risk equation in the data and successfully generated almost identical parameters as those generated from the derivation cohort (Table 2). Overall, the trends in the results presented below were similar between males and females, although the magnitude of effect differed reflecting the differences in starting baseline values seen in Table 1.

**Table 2:** Values of actual risk equation relating BMI to probabilities of developing diabetes using logistic regression values from the National Population Health Survey (NPHS) 10-year follow-up cohort and values achieved from the simulation model

	**Males – NPHS data(N = 9,177)**			**Males – Simulation(N = 9,177)**		
**Variable**	**Coefficient**	**Standard error**	**P-value**	**Coefficient**	**Standard error**	**P-value**
BMI	0.4202	0.0383	< 0.0001	0.4263	0.0111	< 0.0001
BMI^2^	−0.00437	0.000618	< 0.0001	−0.00448	0.001049	< 0.0001
Intercept	−10.4034			−10.4897		
Model properties						
Calibration (χ^2^_HL_)	χ^2^_HL_ = 5.67, p-value = 0.6841			χ^2^_HL_ =9.951, p-value = 0.3689		
Discrimination (C-statistic)	C = 0.677			C = 0.686		
	**Females – NPHS data(N = 10,618)**			**Females – Simulation(N = 10,618)**		
**Variable**	**Coefficient**	**Standard error**	**P-value**	**Coefficient**	**Standard error**	**P-value**
BMI	0.4565	0.0554	< 0.0001	0.4593	0.0779	< 0.0001
BMI^2^	−0.00509	0.00091	< 0.0001	−0.00514	0.00141	< 0.0001
Intercept	−10.8967			−10.5899		
Model properties						
Calibration (χ^2^_HL_)	χ^2^_HL_ = 9.33, p-value = 0.3153			χ^2^_HL_ = 10.466, p-value = 0.3356		
Discrimination (C-statistic)	C = 0.726			C = 0.718		

**Figure 1:**
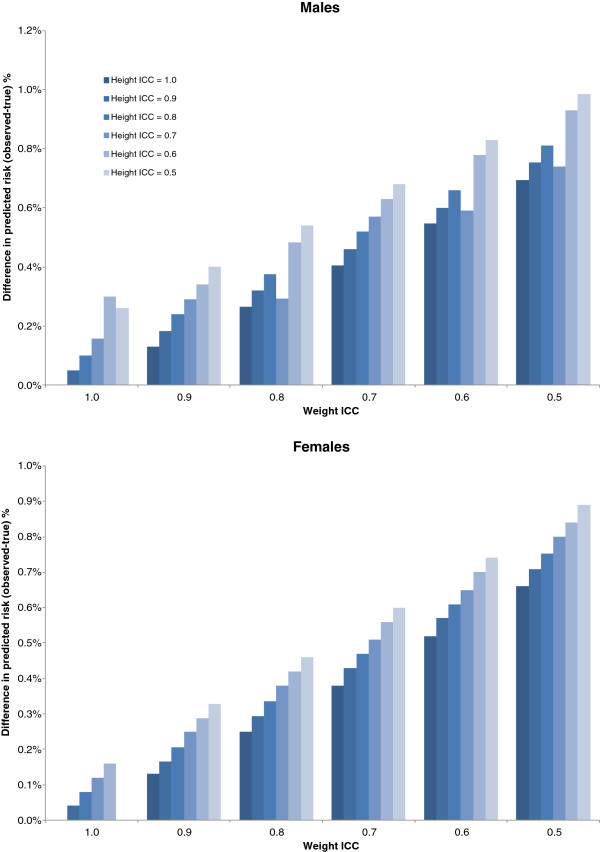
Difference in overall diabetes risk (observed – true) under random error in height and weight for males (N = 9,177) and females (N = 10,618) averaged over 500 replications.

Under the presence of random error, the average values of the observed BMI were similar to the true values; however, the variance was increased. For example, in females when the ICC of height and weight were set to 0.8, the observed mean BMI +/− standard deviation (SD) was equal to 24.47 +/− 4.51 compared to the true value of 24.44 +/− 3.87. The probability of developing diabetes predicted from the risk algorithm under the presence of random error was higher than the estimate applied to the data without random error. In other words, the presence of random error biased the overall predicted risk estimate upwards. The differences between the predicted risk with and without error were relatively small, with the biggest differences being 0.99% higher than the true value, which would predict 90 more diabetes cases for males, and 0.89% higher for females, resulting in the prediction of 95 more diabetes cases for females. Random error in weight had a bigger influence on the predicted risk than error in height. When the ICC for weight was held at 1.0 (i.e., no error) but ICCs in height were allowed to vary (from 0.5 to 1.0), the largest overestimate in diabetes risk was 0.30% in males (28 more diabetes cases) and 0.16% in females (70 more diabetes cases). When the ICC for height was held at 1.0 and the ICCs for weight were allowed to vary, the largest overestimate was 0.70% (64 more cases) for females and 0.66% (39 more cases) for males (Figure 1). The distribution of predicted risk has a narrower range relative to the observed distributions and this influences the right side of the distribution. For example, when systematic error was 0 and ICC was 0.6, the 10-year diabetes risk in females ranges from 0.04% to 33.49% with a 90^th^ percentile equal to 7.0%. In contrast, the distribution based on the true BMI values ranges from 0.01% to 31.98% with a 90th percentile equal to 6.85%.

**Figure 2:**
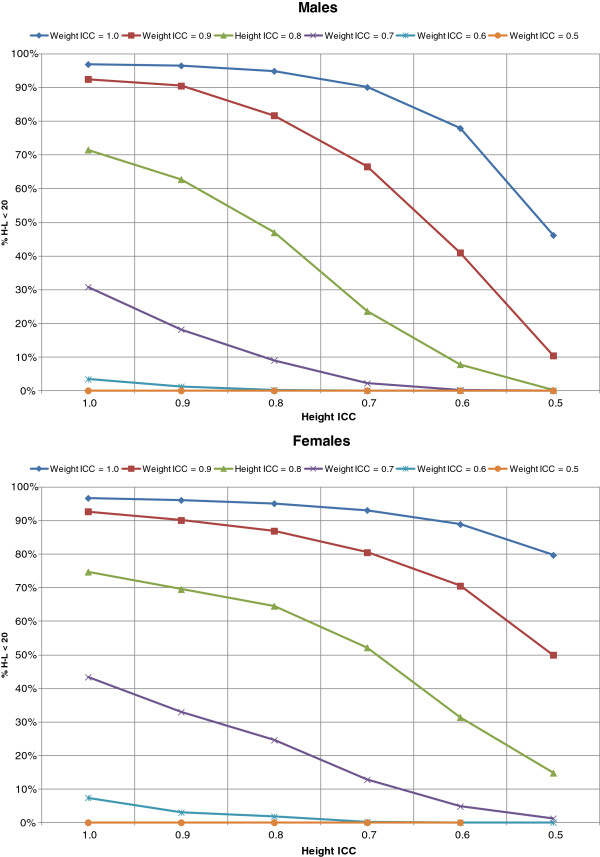
Percent that achieved calibration (H-L ぽχ2 <20) in 500 replications under various levels of random error indicated by the Interclass Correlation Coefficient (ICC) for height and weight among males (N = 9,177) and females (N = 10,618).

When random error was assumed to be absent, the calibration cutoff (χ^2^_H-L_ < 20) was achieved 97% of the time. With increasing levels of random error, the proportion of simulations where the calibration cutoff was achieved decreased steadily. Overall, ICCs of approximately 0.8 or higher resulted in the algorithm achieving the calibration criteria at least 80% of the time. In both males and females, errors in weight lead to larger decreases in calibration than height, such that even a perfect height measurement (ICC height = 1.0) would fail to achieve the calibration cutoff if the ICC for weight drops below 0.8. On the other hand, if ICC for weight was 1.0, even if the ICC for height was 0.6, the algorithm could still achieve calibration almost 80% of the time (Figure 2).

**Figure 3:**
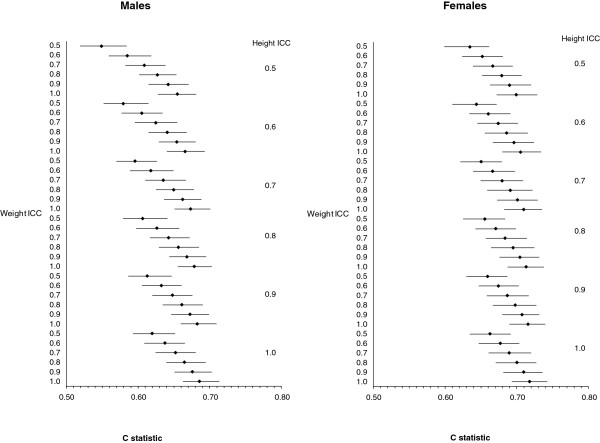
Average C-statistic for 500 replications under random error in height and weight for males (N = 9,177) and females (N = 10,618) indicated by the Interclass Correlation Coefficient (ICC) averaged over 500 simulations.

Discrimination was decreased in the presence of random error. Under the most severe measurement error, the C-statistic was reduced from 0.69 (with no error) to 0.55 in males and from 0.72 (with no error) to 0.63 in females. If the ICCs for height and weight were higher than 0.8, then the differences in the C-statistic compared to the estimate that had no random error were less than 0.02. As with calibration, error in weight had a bigger impact on the C-statistic than errors in height (Figure 3).

**Figure 4:**
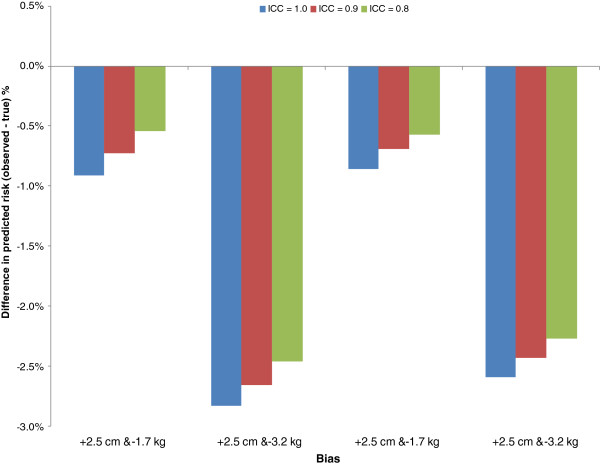
Difference in overall risk (observed Î true) under both systematic error and random error in height and weight among males (N = 9,177) averaged over 500 simulations.

Under systematic error, the observed BMI on average was higher or lower than the true BMI depending on the nature of the directional error. For example, when weight is underestimated by 3.0 kg then the observed mean BMI +/− SD is equal to 24.47 +/− 4.51 compared to the true BMI of 25.61 +/− 4.52, a difference of 1.15 kg/m^2^. As expected, underreporting of weight and overreporting of height resulted in an underestimate of predicted probability of developing diabetes. The average level of systematic error found in the systematic review was an underreporting of weight of 1.7 kg and an overreporting of height of 2.5 cm [29], and at these levels risk would be underestimated by 0.86% (91 fewer cases) in males and a 0.91% reduction (84 fewer cases) in females (Figure 4). The presence of random error in conjunction with systematic error slightly reduced the amount of underestimation.

Overall, underreporting of weight of 1.5 kg or greater, or overreporting of height of 1.5 cm or greater, resulted in the failure of the algorithm achieving the benchmark calibration value at least 80% of the time (Table 3). None of the 500 simulations achieved calibration under the maximum biases in reported height and weight in both males and females (Figure 4). It must be noted that there is no evidence from the literature that these extreme biases are likely in self-reported height and weight; rather, they were investigated to illustrate the range of results of under- and overreporting. The presence of random error in conjunction with systematic error did not significantly worsen or improve power to detect calibration.

**Table 3:** Difference in overall diabetes risk (observed – true) and percent that achieved calibration (H-L χ2 <20) in 500 replications under systematic reporting error (bias) in height and weight for males (N=9,177) and females (10,618)

	**Males (N=9,177)**			**Females (N=10,618)**		
**Reporting bias**	**Difference in diabetes risk (observed - true)**	**Number of diabetes cases**	Percent that achieved calibration*	**Difference in diabetes risk (observed - true)**	**Number of diabetes cases**	**Percent that achieved calibration**
Underestimate of weight by						
0.5 kg	−0.23%	24	95.4%	−0.22%	23	93.0%
1.0 kg	−0.46%	49	87.2%	−0.44%	47	80.2%
1.5 kg	−0.69%	73	73.0%	−0.67%	71	58.6%
2.0 kg	−0.94%	100	49.0%	−0.90%	95	32.2%
2.5 kg	−1.18%	125	23.2%	−1.13%	120	11.8%
3.0 kg	−1.43%	152	7.4%	−1.37%	145	2.0%
Overestimate of height by						
0.5 cm	−0.22%	23	95.4%	−0.19%	20	93.4%
1.0 cm	−0.45%	48	87.4%	−0.38%	40	85.6%
1.5 cm	−0.69%	73	75.0%	−0.57%	61	69.4%
2.0 cm	−0.92%	98	52.4%	−0.77%	82	48.2%
2.5 cm	−1.16%	123	25.8%	−0.98%	104	25.8%
3.0 cm	−1.41%	150	9.4%	−1.19%	126	9.2%

*(H-L χ^2^ <20).

**Figure 5:**
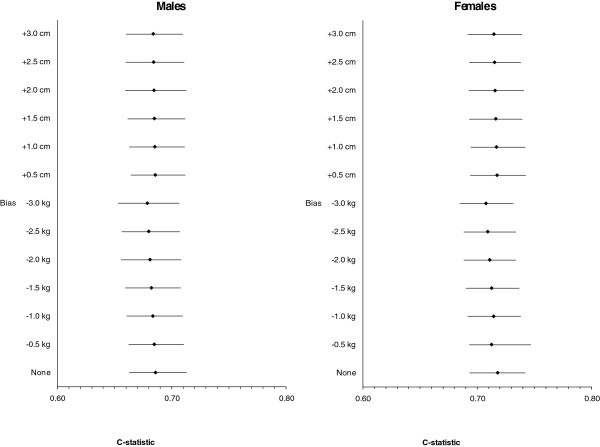
Average C-statistic for 500 replications under systematic reporting error (bias) in height and weight for males (N = 9,177) and females (10,618) indicated by the Interclass Correlation Coefficient (ICC) averaged over 500 simulations.

Under- or overreporting weight and height did not have a significant effect on the discrimination of the model (Figure 5). C-statistics were reduced very slightly in the most extreme case of underreporting of weight or overreporting of height (≤ 0.01 for both males and females). When both random error and systematic error were imposed, the C-statistic was reduced; however, this was due to the influence of random error and not systematic error.

## Discussion

This study systematically examined the impact of measurement error in the context of a prediction algorithm. This simulation study reveals several interesting aspects of the influence of measurement error (systematic and random) on the performance of a risk algorithm.

As hypothesized, random error reduced calibration and discrimination of the algorithm due to the fact that the observed variance is greater than the true variance in the presence of measurement error. The observed BMI distribution was wider than the true distribution due to this increased variation. This affects both diabetics and nondiabetics due to its random nature, resulting in greater overlap between the BMI distributions. Ultimately, this makes assigning risk according to BMI levels more difficult to achieve. Even though random error in height and weight should, on average, correctly estimate the true BMI in the population (since it does not skew the mean in a particular direction), it can still influence the performance of a prediction model due to decreased precision, which leads to greater dispersion in the BMI distribution.

In this study, systematic error in height and weight biased the predicted risk estimates in the direction of the error. This affects calibration, which is not surprising since the concordance of observed and predicted events would be influenced by the under- or overreporting of the BMI level. In other words, persons that are over- or underreporting their weight will then be over- or underestimated by the risk model and thus result in disagreement with observed estimates. Systematic error did not influence the ability to rank order subjects. Therefore, the ability to discriminate between who will and will not develop diabetes was not affected by systematic error when variance due to random error is held constant. This was reflected by the stability of the C-statistic under varying degrees of systematic error. The way that the systematic error was examined in this study was such that the distribution of BMI was shifted to the left (as a result of underestimating weight or overestimating height, or both) compared to the true distribution. This is an overall effect, and the decreased precision or increased variability as seen with random error is therefore not observed. Even though the distribution is shifted to the left, those with higher BMI still have a higher probability of developing diabetes compared to those with lower BMI despite the fact that the absolute levels of risk will be underestimated in both groups. This is a classic example of how discrimination and calibration are often discordant. Due to the nature of probability, it is possible for a prediction algorithm to exhibit perfect discrimination, i.e., it can perfectly resolve a population into those who will and will not experience the event, and at the same time have deficient accuracy (meaning that the predicted probability of developing diabetes does not agree with the true probability) [30]. This study did not impose systematic error with respect to disease status, but it could be hypothesized that if the systematic error were differential between diabetics and nondiabetics that this could indeed affect discrimination.

The finding that random error resulted in the overall predicted risk estimated to be biased upwards was contradictory to the hypothesis that only systematic error will bias the risk estimate. Random error increases the variability of a measurement and increases the range of predicted risk, which is bounded by 0 in the logistic model. In a situation where the outcome probabilities are very high, the skew would be expected to be in the opposite direction. Not surprisingly, the error in predicted risk resulting from underreporting weight or overreporting height is in the anticipated direction (i.e., if weight is underreported the observed risk will be underestimated). Furthermore, the addition of random error to this type of systematic error slightly reduced the amount of underestimation because the random and systematic errors worked in opposite directions. In another situation, random error could potentially augment the error in predicted risk. Such would be the case if systematic error tended to result in an overestimate of risk.

This study shows that random error, which accounts for 20% of the total observed variance (ICC of 0.8 or higher), is unlikely to affect the performance or validation of a prediction model. Research shows that the random error in height and weight reporting is unlikely to exceed that amount [31]. Interestingly, the effects on the predicted diabetes risk were relatively minor, even in situations of high under- or overreporting of weight and height. This is likely because BMI has such a strong relationship with diabetes such that increased risk is apparent even with significant underestimation. The true distributions of BMI in diabetics and nondiabetics are so distinct that even in the presence of underreporting these populations have dissimilar risk for developing diabetes. Had this misclassification affected a variable that did not have such a strong relationship with the outcome, the effect on predicted risk may have been more severe. Furthermore, in this study systematic error in self-reported height and weight was taken as an overall effect in the population. If self-reporting error were significantly more likely to occur in those who were more likely to develop diabetes, then the impact of this bias could be augmented.

This study focused on the overall trend of self-reporting error seen in several validation studies, that is an underestimation of weight and an overestimation of height [29]; however, these patterns may also vary across subpopulations, such as gender and socioeconomic status. Generally, women tend to underestimate weight more so than men, and men tend to overestimate height more so than women [31,32]. Socioeconomic status has been shown to modify these associations such that those of lower socioeconomic status may actually overestimate their weight and/or underestimate their height [33,34]. These subgroups may also have differential diabetes risk and the extent to which this error influences population risk prediction is a topic of future research. In addition, values from a given individual in the population may exceed the maximum values included in this study; however, the influence of this would be more relevant for individual risk prediction tools versus for population prediction.

There are several limitations to consider in the context of this study. Conclusions drawn from this simulation study will relate only to the scenarios simulated and may not apply to all risk algorithm situations. Simulation programs that reflect the specific study conditions to which a study is applied must be created to make conclusions applicable. Another caution in interpreting the findings of this study is that models examined in this exercise are simpler than complicated multivariate risk algorithms encountered in practice. This simpler model allows us to focus on the height and weight error, which is the greatest potential source of error in DPoRT. It should be noted that one of the assumptions of this study is that the only sources of error are in self-reported height and weight. Other sources of error, including error in diabetes status and selection bias in the survey or in sampling, are assumed to be absent.

This study provides novel information about the influence of measurement error in a risk prediction model. By understanding the consequences of measurement error on prediction and algorithm performance, efforts can be made to correct for these errors and thus improve the accuracy and validity of a risk algorithm. Further, efforts must be made to understand the nature of error in self-reporting measurements. Ongoing work to improve the quality of measurements used in risk algorithms will improve model performance. Researchers developing and validating risk tools must be aware of the presence of measurement error and its impact on the performance of their risk tools.

### Ethical approval

The study was approved by the Research Ethics Board of Sunnybrook Health Sciences Centre, Toronto, Ontario, Canada.

## Competing interest

The authors declared that they no competing interest.

## Authors’ contributions

LR carried designed the study, carried out all analyses, and drafted the manuscript. PC provided input into the design of the simulation study including coding and independent verification of the simulation, TS provided statistical advice into the design and interpretation of the results, CM provided advice into the design of the study and the measurement scenarios investigated, JH provided clinical input related to diabetes and risk factors, and DM supervised the study, provided input into the design of the simulation, and helped draft the manuscript. All authors read and approved the final manuscript.
